# Rock Art at the Pleistocene/Holocene Boundary in Eastern South America

**DOI:** 10.1371/journal.pone.0032228

**Published:** 2012-02-22

**Authors:** Walter A. Neves, Astolfo G. M. Araujo, Danilo V. Bernardo, Renato Kipnis, James K. Feathers

**Affiliations:** 1 Laboratório de Estudos Evolutivos Humanos, Instituto de Biociências, Universidade de São Paulo, CP11461, São Paulo, São Paulo, Brazil; 2 Museu de Arqueologia e Etnologia, Universidade de São Paulo, São Paulo, São Paulo, Brazil; 3 Scientia Consultoria, São Paulo, São Paulo, Brazil; 4 University of Washington Luminescence Dating Laboratory, University of Washington, Seattle, Washington, United States of America; University of Oxford, United Kingdom

## Abstract

**Background:**

Most investigations regarding the First Americans have primarily focused on four themes: when the New World was settled by humans; where they came from; how many migrations or colonization pulses from elsewhere were involved in the process; and what kinds of subsistence patterns and material culture they developed during the first millennia of colonization. Little is known, however, about the symbolic world of the first humans who settled the New World, because artistic manifestations either as rock-art, ornaments, and portable art objects dated to the Pleistocene/Holocene transition are exceedingly rare in the Americas.

**Methodology/Principal Findings:**

Here we report a pecked anthropomorphic figure engraved in the bedrock of Lapa do Santo, an archaeological site located in Central Brazil. The horizontal projection of the radiocarbon ages obtained at the north profile suggests a minimum age of 9,370±40 BP, (cal BP 10,700 to 10,500) for the petroglyph that is further supported by optically stimulated luminescence (OSL) dates from sediment in the same stratigraphic unit, located between two ages from 11.7±0.8 ka BP to 9.9±0.7 ka BP.

**Conclusions:**

These data allow us to suggest that the anthropomorphic figure is the oldest reliably dated figurative petroglyph ever found in the New World, indicating that cultural variability during the Pleistocene/Holocene boundary in South America was not restricted to stone tools and subsistence, but also encompassed the symbolic dimension.

## Introduction

In the last few decades, information on the biology and archaeological context of the first Native Americans has greatly increased. For example, we now know that the first Americans looked very different from Late Prehistoric and current Native Americans [1]–[4], that the lithic industry and subsistence pattern of the pioneers varied considerably from region to region [5]–[7], and that people were present in the New World prior to Clovis [8]–[10]. However, little is known about early American art. There are very few cases of rock art in the Americas whose ages can be reliably placed even near the Pleistocene/Holocene boundary [11], [12]. We report here on early rock art discovered at Lapa do Santo, an archaeological site located in Central Brazil.

### Lapa do Santo Rock shelter

Lapa do Santo (19°28′40″S, 44°02′20″W) is a limestone rock shelter located in the northern sector of the Lagoa Santa Karst, approximately 60 kilometers from Belo Horizonte, in Central-Eastern Brazil (Figure S1). The site is one of the largest rock shelters excavated in the Lagoa Santa region, with a sheltered area of 1,300 m^2^ (70 meters long and 20 meters wide). Excavations at Lapa do Santo were conducted between 2002 and 2009. An area of 44 m^2^ (Figure S2) was excavated following natural levels.

The sedimentary matrix is mainly composed of wood ashes derived from hearths established within the rock-shelter over the last 12.0 kyr. Geogenic sediments contributed very little to the formation of the matrix. The depth of the archaeological sediment varies from a few centimeters to almost 4.0 meters. Sixty-two radiocarbon ages were generated for Lapa do Santo, either on charcoal samples or on human bones (Table S1). These ages demonstrate that the site was occupied during three different periods: one spanning from circa 12 kyr BP (10,000 ^14^C yr BP) to 8 kyr BP (7,500 ^14^C yr BP), a second around 4.4 kyr BP (4,000 ^14^C yr BP), and a third, around 0.8 kyr BP (900 ^14^C yr BP). This pattern of occupation is in agreement with data from other rock-shelters excavated at Lagoa Santa [13]. While the earliest occupation covered most of the habitable area of the site, the two later occupations were restricted to the southern portion of the sheltered area.

The lithic industry can be characterized as expedient, with very few formal artifacts. Most tools are represented by small flakes with little investment in marginal retouch. Notwithstanding, a few bifacial point pre-forms were recovered during the excavations. The Lagoa Santa industry bears no resemblance to other contemporaneous Eastern South American lithic traditions [14]. Bone tools, although rare, were also found at Lapa do Santo. They are represented mainly by spatulae and borers. A significant amount of faunal and macro-botanical remains were also recovered, indicating that subsistence was based mostly on small game hunting and fruit gathering, as in other parts of Lowland South America [5], [15]–[16]. A total of 27 human burials were recovered from Lapa do Santo, most of them dated to between 9.5 kyr BP (8,500 ^14^C yr BP) and 8.2 kyr BP (7,500 ^14^C yr BP).

### The pecked anthropomorphic figure engraved in the bedrock

**Figure 1:**
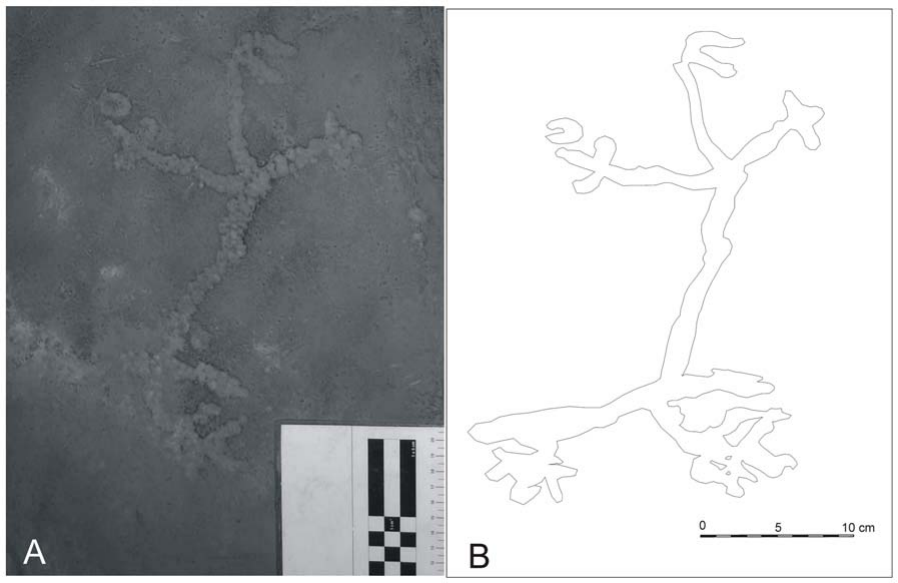
Aspect of the petroglyph. Photograph (A) and contour (B).

In July 2009, during the final days of excavation of unit FG-13/14 in Lapa do Santo, an anthropomorphic figure was exposed at the bottom of the archaeological deposit at an approximate depth of 4.0 meters below the surface. The figure was pecked in the bedrock (Figure 1) and consisted of a small anthropomorphic filiform petroglyph with tri-digits, a c-like head, and an oversized phallus. The figure is 30 cm long (from head to feet) and 20 cm wide. Similar filiform figures can be observed in a niche on the wall of the rock-shelter.

In order to investigate the antiquity of the petroglyph and to discuss its implication in the studies of settlement of New World we analyzed radiocarbon ages and stimulated luminescence dates (OSL) from stratigraphic profile near to the rock art finding.

## Results

**Figure 2:**
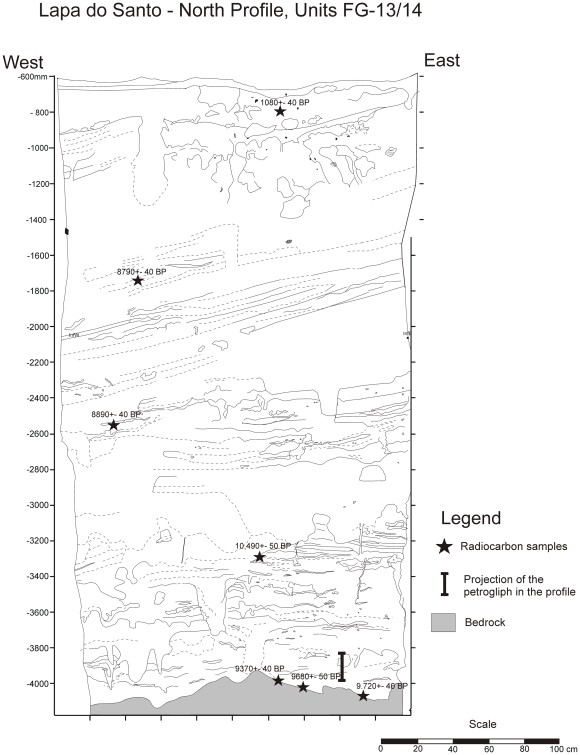
Stratigraphic profile of Unit FG – 13/14. In the profile is shown the radiocarbon ages of charcoal found in the wall and the projection of the petroglyph into the profile. There is only one instance of age inversion, which would put the figure in an even older time interval.

The Lapa do Santo stratigraphy is very well preserved, and instances of sediment mixing are easily recognized due to the structure of the ash layers. Radiocarbon ages are consistent with depth (Figure 2). The horizontal projection of the radiocarbon ages obtained at the north profile suggests a minimum age of 9,370±40 BP, (cal BP 10,700 to 10,500) for the petroglyph. Furthermore, the figure was found approximately 30 millimeters below a hearth dated to 9,470±50 BP (cal BP 11,060 to 10,580). Taking into account radiocarbon ages alone (Table S1), we can confidently state that the petroglyph is older than 10.5 thousand years (kyr), perhaps being as old as 12 kyr, which means that this pecked figure could be the oldest figurative petroglyph ever found in the New World.

**Figure 3:**
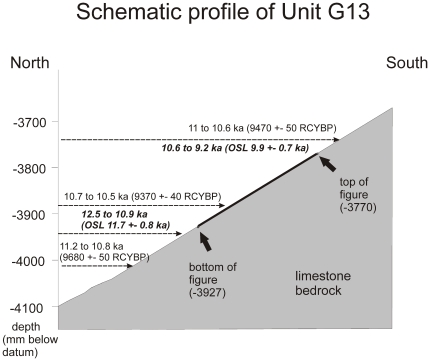
Schematic positioning of the petroglyph. The draw is showing the relative projection of the figure (petroglyph), radiocarbon and luminescence ages.

The antiquity of the petroglyph is further supported by optically stimulated luminescence (OSL) dates from sediment in the same stratigraphic unit that covers the figure. A date of 11.7±0.8 kyr is drawn from dating of 328 grains of 150–180 µm quartz (Table S2). The distribution of equivalent dose values among grains showed an over-dispersion of 30%, but 98% of the grains were consistent with an age of at least 10.2±1.0 kyr. The age of 11.7 kyr was computed using the central age model [17]. The sample is located 9 cm above the bedrock, and about 20 cm below another OSL sample dated to 9.9±0.7 kyr (Figure 3).

## Discussion

Rock-art similar to the one reported here can be found in at least two other rockshelters in the same region, Lapa do Ballet and Lapa das Caieiras. However, the stylistic similarity is not restricted to Lagoa Santa but extends to other parts of Brazil [18]. Two stylistic traditions have been defined in Northeastern Brazil: The Nordeste and the Agreste Traditions. The Nordeste Tradition is indirectly dated to between 12 and 6 kyr, while the Agreste Tradition seems to be later, spanning from 9 to 2 kyr, although there is some controversy about these ages [18]–[20]. The figures and scenes depicted in the Nordeste tradition (especially those of the Seridó Sub-Tradition, in Rio Grande do Norte state) are very similar to those found at Lapa do Santo, Lapa do Ballet and Lapa das Caieiras, in Lagoa Santa (Figure S3) [21], [22]. This suggests cultural contact among groups as far apart as 1,600 km by the beginning of the Holocene in Eastern South America.

Baixão do Perna I site, in Piauí State, Brazil, has been considered one of the oldest reliably dated rock art in the Americas [23] with an age of 10.8 kyr (9,540±170 ^14^C yr BP, GIF 5414 [24]). However, the age of the paintings was based on a correlation to a layer of charcoal associated with human occupation, not directly placed over the figures. In Oregon State, USA, geometric petroglyphs were found partially buried by Mount Mazama ash [25], which is dated to 7.7 kyr [26]. Rock art showing mammoth-like figures are present in the Great Basin and in the Colorado Plateau of the USA, also suggesting great antiquity, although no direct dating was possible [27], [28]. Mud Portage site, in Canada, showed a rock pavement with petroglyphs, and covered by archaeological sediments dating between 5 kyr and 9 kyr [29], [30]. In Argentina, linear marks were found in the bedrock of Epullán Grande cave [11], partially covered by archaeological sediments, with a minimum age of 11.6 kyr (9,970±100 ^14^C yr BP). However, there is a pending controversy about the anthropic origin of the marks [31]. In this context, the petroglyph found at Lapa do Santo is the oldest, indisputable testimony of rock art in the Americas.

Several authors have suggested that a short chronology for the occupation of the New World cannot account for the variability of the South American lithic industries in the Early Holocene [14], [32]–[34]. When the data presented here are compared to other examples of Early Holocene rock-art in South America, a scenario of high variability also emerges. For instance, in Argentina, at Cueva de las Manos, contoured hands predominate in the panels; at Cueva Epullan Grande, only geometric motifs were engraved. In Brazil, painted anthropomorphs were predominant at Baixão do Perna I. These data strongly suggest that cultural variability during the Pleistocene/Holocene boundary in South America was not restricted to stone tools and subsistence, but also encompassed the symbolic dimension. Both pieces of information converge to support a deep chronology for the peopling of the New World [10].

## Materials and Methods

OSL ages were obtained by single-grain dating. About 1,100 grains in the 150–180 µm range were measured for equivalent dose using a 540 nm laser on the single-grain apparatus of a Risø DA-TL-15 reader, using procedures similar to those reported elsewhere [10], [35]. Of the 1,100 grains, only 328 produced suitable results (Table S2), the others being rejected based on various criteria [33], mainly due to a lack of measurable signal. A test comparing preheats of 180°C and 240°C produced no discernable difference, so a preheat of 240°C was employed for most grains. A dose recovery test, using an administered dose of 300 s beta irradiation, produced an “equivalent dose” of 300±16 s beta, with an over-dispersion of 16%, using the central age model of Galbraith et al. [17].

The over-dispersion in equivalent dose of the 328 grains used for dating is 30%. Because 16% can be attributed to internal sources of error (from the dose recovery test where the given dose is known), the other 14% is due to external factors. Some of this may be due to differential dose rates, but some could be due to mixing different aged grains. Using a finite mixture model, 98% of the grains can be attributed to components that give an age of at least 10.2±1.0 kyr. This suggests mixing is fairly minimal and localized. The age of 11.7±0.8 kyr is based on the central age model. The sample is also located beneath another sample, which gives an age of 9.9±0.7 kyr and which had an over-dispersion of only 19%, suggesting very little mixing. The dose rate was determined on the bulk sample by alpha counting, beta counting and flame photometry. Results from these were comparable to dose rates determined by a CaSO_4_:Dy dosimeter placed in the field.

## Supporting Information

Figure S1 **Location of Lapa do Santo in Eastern South America.**
(TIF)

Figure S2 **Lapa do Santo rockshelter topography and schematic sections.**
(TIF)

Figure S3 **Examples of C-shaped head anthropomorphs:** A) Lapa do Ballet - MG [21]; B) Lapa das Caieiras - MG; C) Carnaúba dos Dantas - RN [22].
(TIF)

Table S1 **Radiocarbon ages for Lapa do Santo.**
(DOC)

Table S2 **OSL date for Lapa do Santo – Sample UW 1374.**
(DOC)
