# Supplementary figures and images for: Rock Art at the Pleistocene/Holocene Boundary in Eastern South America
Source: PLoS One. 2012 Feb 22;7(2):e32228. doi: 10.1371/journal.pone.0032228 (PMC3284556; doi:10.1371/journal.pone.0032228)

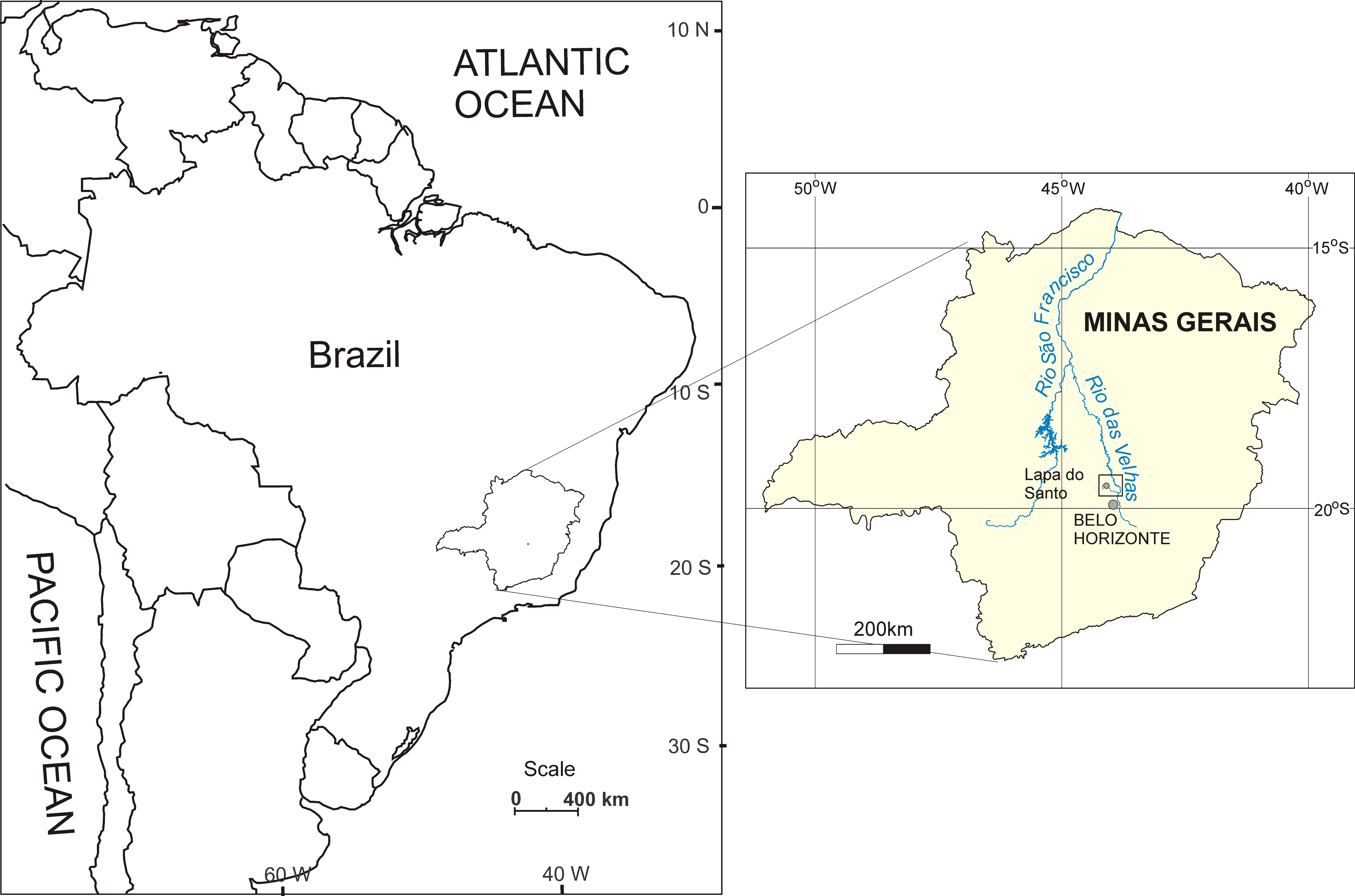

Supplement: Figure S1 — Location of Lapa do Santo in Eastern South America. (TIF) [file pone.0032228.s001.tif]

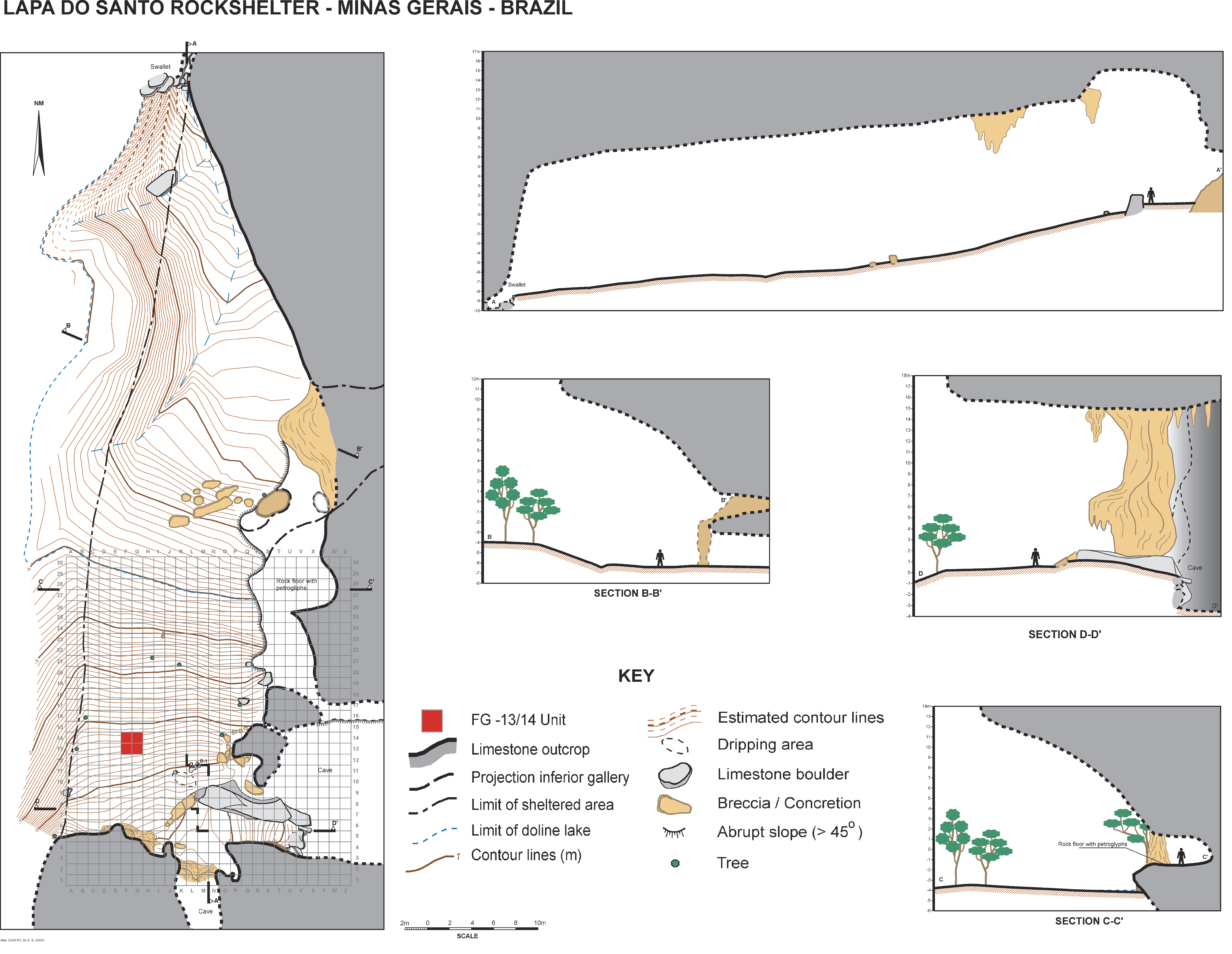

Supplement: Figure S2 — Lapa do Santo rockshelter topography and schematic sections. (TIF) [file pone.0032228.s002.tif]

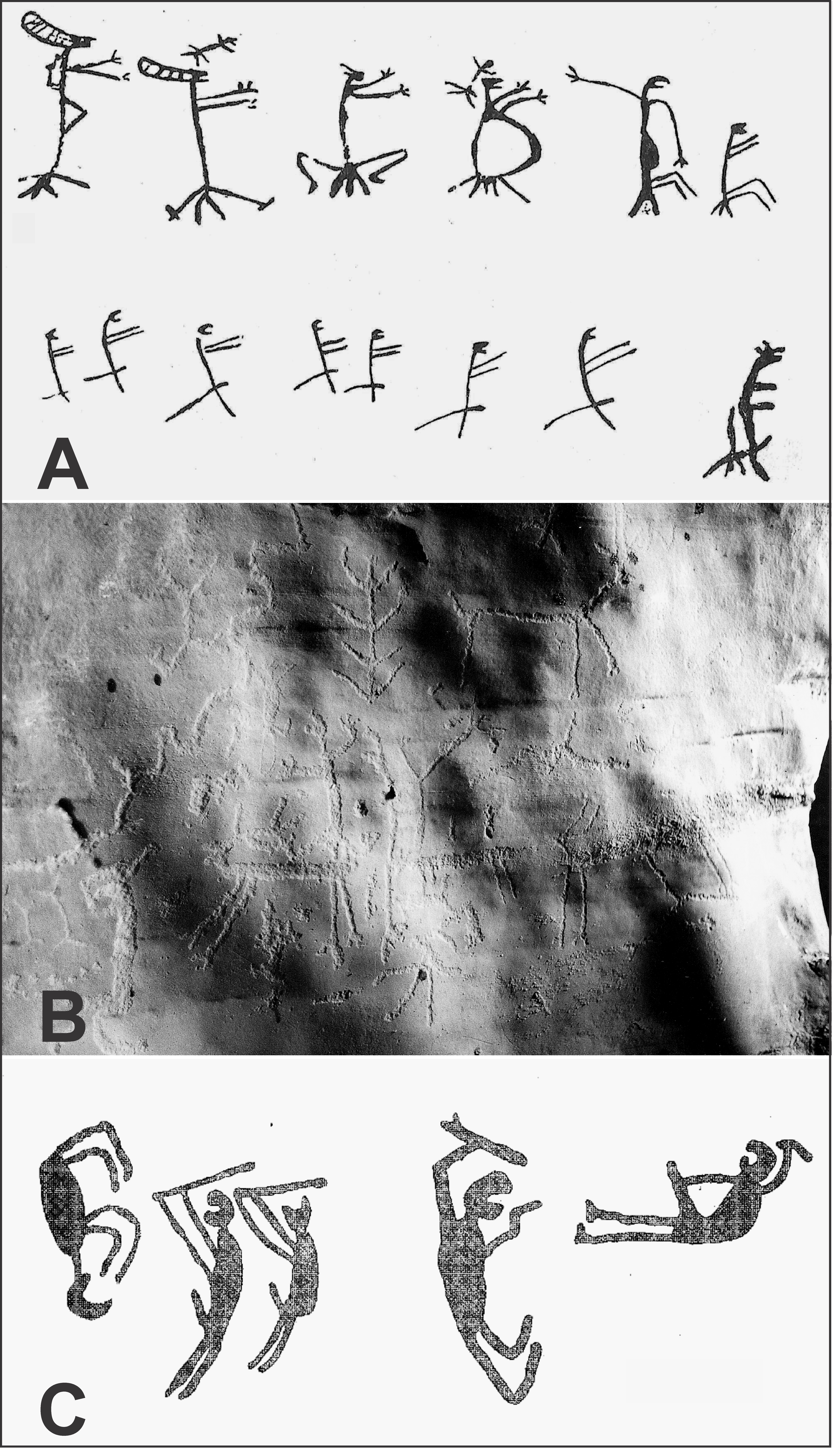

Supplement: Figure S3 — Examples of C-shaped head anthropomorphs: A) Lapa do Ballet - MG [21]; B) Lapa das Caieiras - MG; C) Carnaúba dos Dantas - RN [22]. (TIF) [file pone.0032228.s003.tif]
