# Supplementary material for: Rock Art at the Pleistocene/Holocene Boundary in Eastern South America
Source: PLoS One. 2012 Feb 22;7(2):e32228. doi: 10.1371/journal.pone.0032228 (PMC3284556; doi:10.1371/journal.pone.0032228)
Supplement: Table S1 — Radiocarbon ages for Lapa do Santo. (DOC) [file pone.0032228.s004.doc]

| Sample | Lab number (Beta) | Material | Conventional ^14^C age  (years B.P.) | ^13^C / ^12^C ratio | Calibration 2 sigma * |
| --- | --- | --- | --- | --- | --- |
| Burial 2 | 253497 | Human bone | 790 ± 40 | -19.2 o/oo | 780 to 670 cal B.P. |
| LST45 | 202764 | charred material | 910 ± 50 | -25.6 o/oo | 930 to 710 cal B.P. |
| ST-47 | 183572 | charred material | 960 ± 70 | -25.0 o/oo | 980 to 720 cal B.P. |
| ST-A1753 | 280491 | charred material | 1,080 ± 40 | -26.6 o/oo | 1,060 to 930 cal B.P. |
| LST59 | 202766 | charred material | 3,810 ± 50 | -26.0 o/oo | 4,400 to 4,080 cal B.P.,  4,030 to 4,010 cal B.P. |
| LST58 | 202765 | charred material | 3,820 ± 100 | -25.7 o/oo | 4,510 to 4,480 cal B.P.,  4,440 to 3,910 cal B.P. |
| ST-10 | 183570 | charred material | 3,830 ± 40 | -25.5 o/oo | 4,400 to 4,100 cal B.P. |
| LSt04 | 216517 | charred material | 3,930 ± 40 | -24.9 o/oo | 4,500 to 4,480 cal B.P.,  4,440 to 4,250 cal B.P. |
| 359 | 243393 | charred material | 3,950 ± 40 | -23.4 o/oo | 4,520 to 4,460 cal B.P.,  4,450 to 4,290 cal B.P. |
| LST6 | 214129 | charred material | 3,960 ± 40 | -26.7 o/oo | 4,520 to 4,290 cal B.P. |
| ST-72 | 183574 | charred material | 4,010 ± 130 | -25.0 o/oo | 4,840 to 4,100 cal B.P. |
| LSt50 | 216521 | charred material | 4,070 ± 60 | -25.0 o/oo | 4,820 to 4,420 cal B.P. |
| 335 | 243392 | charred material | 4,140 ± 40 | -26.3 o/oo | 4,830 to 4,530 cal B.P. |
| LST73 | 202768 | charred material | 4,290 ± 90 | -25.7 o/oo | 5,050 to 4,580 cal B.P. |
| Santo - 1431 | 248891 | charred material | 4,470 ± 40 | -24.5 o/oo | 5,300 to 4,960 cal B.P. |
| Burial 11 | 215195 | Human bone | 5,990 ± 40 | -20.6 o/oo | 6,900 to 6,730 cal B.P. |
| Burial 7B | 215194 | Human bone | 7,400 ± 40 | -18.9 o/oo | 8,330 to 8,160 cal B.P. |
| Burial 19 | 215200 | Human bone | 7,700 ± 40 | -18.6 o/oo | 8,560 to 8,400 cal B.P. |
| LST760 | 214142 | charred material | 7,890 ± 40 | -25.1 o/oo | 8,970 to 8,910 cal B.P., 8,870 to 8,830 cal B.P., 8,790 to 8,590 cal B.P. |
| LSF13NO.16 | 159246 | charred material | 7,940 ± 50 | -26.5 o/oo | 8,990 to 8,630 cal B.P. |
| Santo - 1696 | 248893 | charred material | 8,170 ± 50 | -25.2 o/oo | 9,280 to 9,010 cal B.P. |
| ST-62 | 183573 | charred material | 8,230 ± 50 | -25.8 o/oo | 9,400 to 9,340 cal B.P.,  9,320 to 9,030 cal B.P. |
| Burial 14 | 215196 | Human bone | 8,230 ± 40 | -22.4 o/oo | 9,380 to 9,370 cal B.P.,  9,300 to 9,040 cal B.P. |
| LST63 | 202767 | charred material | 8,530 ± 40 | -26.5 o/oo | 9,550 to 9,490 cal B.P. |
| Burial 26 | 253511 | Human bone | 8,540 ± 50 | -19.8 o/oo | 9,550 to 9,480 cal B.P. |
| Burial 17 | 265182 | Human bone | 8,580 ± 50 | -19.0 o/oo | 9,590 to 9,490 cal B.P. |
| ST-40 | 183571 | charred material | 8,600 ± 160 | -25.0 o/oo | 10,150 to 9,280 cal B.P. |
| LST15 | 202763 | charred material | 8,600 ± 50 | -26.8 o/oo | 9,660 to 9,510 cal B.P. |
| LSt736 | 216523 | charred material | 8,620 ± 40 | -26.0 o/oo | 9,660 to 9,530 cal B.P. |
| Burial 17 | 253507 | Human bone | 8,660 ± 50 | -19.0 o/oo | 9,710 to 9,540 cal B.P. |
| LST89 | 214131 | charred material | 8,670 ± 40 | -26.8 o/oo | 9,720 to 9,540 cal B.P. |
| LSt21 | 216519 | charred material | 8,690 ± 40 | -26.2 o/oo | 9,740 to 9,550 cal B.P. |
| LST100 | 214133 | charred material | 8,700 ± 40 | -26.0 o/oo | 9,760 to 9,550 cal B.P. |
| LSt759 | 216524 | charred material | 8,710 ± 40 | -24.8 o/oo | 9,860 to 9,860 cal B.P.,  9,780 to 9,550 cal B.P. |
| LST726 | 214136 | charred material | 8,710 ± 80 | -25.8 o/oo | 10,110 to 10,080 cal B.P.,  9,930 to 9,530 cal B.P. |
| Burial 14 | 253505 | Human bone | 8,730 ± 50 | -19.6 o/oo | 9,900 to 9,550 cal B.P. |
| LST712 | 214135 | charred material | 8,750 ± 40 | -25.4 o/oo | 9,900 to 9,570 cal B.P. |
| LST94 | 214132 | charred material | 8,790 ± 40 | -24.8 o/oo | 10,120 to 10,070 cal B.P.,  9,920 to 9,670 cal B.P. |
| ST-A1751 | 280490 | charred material | 8,790 ± 40 | -26.2 o/oo | 10,120 to 10,070 cal B.P.,  9,920 to 9,670 cal B.P. |
| LSt71 | 216522 | charred material | 8,800 ± 40 | -26.3 o/oo | 10,120 to 10,070 cal B.P.,  9,940 to 9,690 cal B.P. |
| LSt48 | 216520 | charred material | 8,810 ± 90 | -26.3 o/oo | 10,190 to 9,550 cal B.P. |
| LST731 | 214137 | charred material | 8,820 ± 40 | -26.3 o/oo | 10,140 to 10,000 cal B.P.,  9,960 to 9,710 cal B.P. |
| Sep 1 | 271249 | Human bone | 8,840 ± 60 | -18.1 o/oo | 10,180 to 9,680 cal B.P.,  8,230 to 7,730 cal B.P. |
| LST711 | 214134 | charred material | 8,870 ± 100 | -24.1 o/oo | 10,220 to 9,570 cal B.P. |
| ST-A1734 | 280487 | charred material | 8,890 ± 40 | -24.2 o/oo | 10,180 to 9,890 cal B.P. |
| LSF13NO.17 | 159247 | charred material | 8,880 ± 50 | -26.4 o/oo | 10,190 to 9,770 cal B.P. |
| 364 | 246246 | charred material | 8,900 ±40 | -24.9 o/oo | 10,190 to 9,900 cal B.P. |
| LST738 | 214139 | charred material | 8,930 ± 40 | -25.9 o/oo | 10,200 to 9,920 cal B.P. |
| LST744 | 214140 | charred material | 8,930 ± 40 | -25.2 o/oo | 10,200 to 9,920 cal B.P. |
| LST751 | 214141 | charred material | 8,980 ± 40 | -26.1 o/oo | 10,220 to 10,140 cal B.P., 10,000 to 9,960 cal B.P. |
| LSt19 | 216518 | charred material | 9,100 ± 40 | -24.1 o/oo | 10,260 to 10,200 cal B.P. |
| LST762 | 214143 | charred material | 9,150 ± 40 | -24.8 o/oo | 10,400 to 10,220 cal B.P. |
| ST-A1740 | 263885 | charred material | 9,370 ± 40 | NA | 10,700 to 10,500 cal B.P. |
| ST-A1737 | 263883 | charred material | 9,470 ± 50 | NA | 11,060 to 11,020 cal B.P., 11,010 to 10,960 cal B.P.,  10,800 to 10,580 cal B.P. |
| LS1475 | 256223 | charred material | 9,520 ± 60 | -24.9 o/oo | 11,150 to 10,550 cal B.P. |
| ST-A1724 | 263882 | charred material | 9,650 ± 50 | -25.6 o/oo | 11,200 to 11,050 cal B.P.,  11,040 to 10,780 cal B.P. |
| ST-A1738 | 263884 | charred material | 9,680 ± 50 | -23.1 o/oo | 11,210 to 11,070 cal B.P., 10,960 to 10,860 cal B.P.,  10,840 to 10,810 cal B.P. |
| ST-A1739 | 280488 | charred material | 9,720 ± 40 | 22.2 o/oo | 11,220 to 11,100 cal B.P. |
| LST77 | 214130 | charred material | 9,900 ± 40 | -23.7 o/oo | 11,320 to 11,210 cal B.P. |
| LST734 | 214138 | charred material | 10,070 ± 100 | -23.0 o/oo | 12,310 to 11,230 cal B.P. |
| LS1488 | 256224 | charred material | 10,130 ± 60 | -28.0 o/oo | 12,050 to 11,400 cal B.P. |
| ST-A1745 | 280489 | charred material | 10,490 ± 50 | -23.2 o/oo | 12,680 to 12,350 cal B.P.,  12,320 to 12,240 cal B.P. |

* INTCAL 04 Radiocarbon Age Calibration.
