# Supplementary material for: Rock Art at the Pleistocene/Holocene Boundary in Eastern South America
Source: PLoS One. 2012 Feb 22;7(2):e32228. doi: 10.1371/journal.pone.0032228 (PMC3284556; doi:10.1371/journal.pone.0032228)
Supplement: Table S2 — OSL date for Lapa do Santo – Sample UW 1374. (DOC) [file pone.0032228.s005.doc]

| *UW1374* | |
| --- | --- |
| Dose Rate | |
| U (ppm) | 2.72 ± 0.25 |
| Th (ppm) | 17.52 ± 1.66 |
| K (%) | 0.71 ± 0.02 |
| Dose rate (Gy/ka) | 2.07 ± 0.10 |
| Equivalent Dose | |
| Number of measurable grains | 328 |
| D_e_ (Gy) – central age | 24.2 ± 0.5 |
| Over-dispersion (%) | 29.9 ± 1.9 |
| Finite Mixture Model | |
| D_e_ (Gy) – 2^nd^ component (57%) | 21.2 ± 1.5 |
| D_e_ (Gy) – 3^rd^ component (41%) | 31.0 ± 2.2 |
| Age (ka) | |
| Age (2^nd^ component) | 10.2 ± 1.0 |
| Age (3^rd^ component) | 15.0 ± 1.4 |
| Age (central age) | 11.7 ± 0.8 |
